# Meta-Analysis of Microarray Data of Rainbow Trout Fry Gonad Differentiation Modulated by Ethynylestradiol

**DOI:** 10.1371/journal.pone.0135799

**Published:** 2015-09-17

**Authors:** Sophie Depiereux, Florence Le Gac, Bertrand De Meulder, Michael Pierre, Raphaël Helaers, Yann Guiguen, Patrick Kestemont, Eric Depiereux

**Affiliations:** 1 University of Namur (UNamur), Research Unit in Environmental and Evolutionary Biology (URBE-NARILIS), 61 rue de Bruxelles, B-5000, Namur, Belgium; 2 University of Namur (UNamur), Research Unit in Molecular Biology (URBM-NARILIS), 61 rue de Bruxelles, B-5000, Namur, Belgium; 3 Institut National de la Recherche Agronomique, INRA-LPGP, UPR1037, Campus de Beaulieu, 35042, Rennes, France; Universitat de Barcelona, SPAIN

## Abstract

Sex differentiation in fish is a highly labile process easily reversed by the use of exogenous hormonal treatment and has led to environmental concerns since low doses of estrogenic molecules can adversely impact fish reproduction. The goal of this study was to identify pathways altered by treatment with ethynylestradiol (EE2) in developing fish and to find new target genes to be tested further for their possible role in male-to-female sex transdifferentiation. To this end, we have successfully adapted a previously developed bioinformatics workflow to a meta-analysis of two datasets studying sex reversal following exposure to EE2 in juvenile rainbow trout. The meta-analysis consisted of retrieving the intersection of the top gene lists generated for both datasets, performed at different levels of stringency. The intersecting gene lists, enriched in true positive differentially expressed genes (DEGs), were subjected to over-representation analysis (ORA) which allowed identifying several statistically significant enriched pathways altered by EE2 treatment and several new candidate pathways, such as progesterone-mediated oocyte maturation and PPAR signalling. Moreover, several relevant key genes potentially implicated in the early transdifferentiation process were selected. Altogether, the results show that EE2 has a great effect on gene expression in juvenile rainbow trout. The feminization process seems to result from the altered transcription of genes implicated in normal female gonad differentiation, resulting in expression similar to that observed in normal females (*i*.*e*. the repression of key testicular markers *cyp17a1*, *cyp11b*, *tbx1*), as well as from other genes (including transcription factors) that respond specifically to the EE2 treatment. The results also showed that the bioinformatics workflow can be applied to different types of microarray platforms and could be generalized to (eco)toxicogenomics studies for environmental risk assessment purposes.

## Introduction

### 1.1. Transdifferentiation in fish exposed to exogenous hormones

In gonochoristic species, gonads (*i*.*e*. testis or ovary) develop from a germ and somatic cell primordium [1]. Sex determination can be genetically controlled (for instance in XX/XY systems, the expression of Y-linked genes leads to testicular development), and the subsequent differentiation processes are supported by molecular and endocrine factors, such as sex steroid hormones [2]. While these processes are strictly genetically controlled in mammals, where the expression of a main male gene, SRY (Sex-determining Region of Y chromosome), irrefutably leads to testis development, gonadal sex differentiation in fish is more labile, which means that their phenotype may be different from their sex genotype. Based on preliminary studies of steroid treatment influence on fish sex phenotype conducted by Yamamoto in the late 1960s, which highlighted this plasticity, exogenous steroid treatments were used in aquaculture to control the gender of fish stocks [3]. However, although the technique is now widely used, the precise mechanisms underlying this transdifferentiation process are still mostly unknown. In the current environmental context, pollution of rivers with endocrine disrupting compounds (EDCs; molecules that mimic natural endogenous hormones or interfere with the endogenous synthesis of hormones) has given rise to concerns about the sensitivity of fish to exogenous hormones, since very low doses (within ng to μg/L range) of these compounds are able to disrupt gonad morphology, leading to higher rates of intersexuality (gonads displaying male and female features simultaneously) and abnormalities. Several studies have shown that these disruptions could affect the fertility of fish and decrease population fitness [4,5]. Considering that EDCs do not necessarily mimic the endogenous physiological processes [6–8], a clear understanding of these mechanisms is crucial.

### 1.2. Microarray approaches

Modern genomic technologies, such as DNA microarray assays, are powerful tools to investigate initial responses to exogenous agents[9], and a considerable amount of microarray data has been and continues to be generated, for an increasing range of species [10]. The rapid expansion of public genomic databases and the development of bioinformatics tools have considerably enhanced our ability to infer biological meaning from large gene lists. This technique has proven to be efficient for the study of predicted biological pathways and regulatory networks, with wide ranging applications from human clinical biomarker discovery to drug development [11]. More recently, ecotoxicological studies aimed at determining the molecular mode of action (MOA) of environmental pollutants and identifying their biomarkers as indicators of exposure have been published [11,12]. However, certain practices commonly used for human and rodent studies are not fully adapted to the use of microarrays in aquatic ecotoxicology studies. Indeed, the inference of biological meaning from large gene lists relies on the quality of the genomic resources for the species investigated, and especially the annotation of the oligonucleotides spotted and the body of pre-existing genomic knowledge (ontologies, pathways, …). Though the genomes of a growing number of fish species have been sequenced, the available specific genomic resources are limited, hampering data-mining efforts.

Inherent issues related to microarray data analysis must also be taken into account. Even at the lowest significance threshold, thousands of statistical tests inevitably contaminate the differentially expressed gene (DEG) lists with a high number of false positives and false negatives [13]. Many efforts were made in the 2000s to develop new statistical methods [14] improving the gene variance estimation [15–18]. However, the problem still remains fundamentally unsolved, the combination of existing methods generates millions of possibilities [19] and the appropriate statistical analysis strategy is not handled by all experimental laboratories. Therefore, gene top lists obtained after statistical analysis depend on the methodology used and can dramatically differ between research teams.

### 1.3. Re-analysis and Meta-analysis

In view of the considerations listed above, most microarray datasets remain underexploited. However, the fast evolution of (i) available data in public genomic databases, (ii) statistical analysis performances and (iii) data-mining bioinformatics tools has made it possible to gain new information through the re-analysis of previously published datasets. Moreover, the meta-analysis of several datasets is a way to yet further enhance analytical performance [19–21]. In a previous work, we successfully adapted a microarray analysis workflow validated on human cancer data [19,22][23] to a toxicogenomic study using rainbow trout as model organism [24]. We measured gene expression in juvenile male fish exposed chronically to several doses of the potent xenoestrogen ethynylestradiol (EE2). This allowed highlighting several pathways and ontologies related to EE2 exposure and to propose several potential specific and/or sensitive biomarkers of the intersex stage in juvenile rainbow trout. Here, we propose to apply this methodology to the re-analysis of a previously published dataset studying the effect of EE2 on a time-course experiment using juvenile male rainbow trout. We postulate that the meta-analysis of both datasets will allow highlighting the pathways and key genes potentially implicated in early male-to-female gonadal transdifferentiation.

## Methods

Two datasets were used in the meta-analysis. The microarray analysis workflow is explained below and summarized in Table 1.

**Table 1:** Summary of the meta-analysis workflow.

	Dataset 1	Dataset 2
**Species**	*Oncorhynchus mykiss*—Genetically all-male population	*Oncorhynchus mykiss*—Genetically all-male and all-female populations
**Treatment conditions**	Balneation—EE2–5 concentrations : 0 (control); 0.01 ; 0.1 ; 1 and 10 μg/L	Feeding—EE2–1 concentration : 20 mg/kg food
**Developmental stage**	Day 0 (D0) at 60 dpf (first feeding) up to 136 dpf	D0 at 55 dpf (first feeding) up to 111 dpf
**Sampling**	One sampling at 136 dpf	7 samplings at different developmental stages: D0, D0+7 days (D7), D16, D30, D63, D91, and D111
**Sample size**	Pools of gonads from ten fish per sample. 6 replicates per condition	Pools from 20 to 100 fish per sample. 2 replicates per condition
**Platform used**	8x60K oligonucleotide array (Agilent technology)	9,023 trout cDNA platform
**Statistical analysis**	One-way ANOVA, with EE2 concentration as criterion	Two-way ANOVA with time-course (D0 to D111) and sex as criteria
**Scheffe’s contrasts**	**4 physiologically relevant contrasts defined as control vs. different EE2 concentrations:** (1) Control vs. 0.01 μg/L—CT1; (2) Control vs. 0.1 μg/L—CT2; (3) Control vs. 1 μg/L—CT3; (4) Control vs. 10 μg/L—CT4	**19 physiologically relevant contrasts defined as Male-EE2 (= male test = MT) versus male-control (MC) at the same sampling time:** (1) MT-MC D7, (2) MT-MC D16, (3) MT-MC D30, (4) MT-MC D63, (5) MT-MC D91, (6) MT-MC D111; **Male-EE2 (= male test = MT) versus female-control (FC) at the same sampling time:** (7) MT-FC D7, (8) MT-FC D16, (9) MT-FC D30, (10) MT-FC D63, (11) MT-FC D91, (12) MT-FC D111; **Male-control (MC) versus female-control (FC) at the same sampling times**: (13) MC-FC D0, (14) MC-FC D7, (15) MC-FC D16, (16) MC-FC D30, (17) MC-FC D63, (18) MC-FC D91, (19) MC-FC D111.
**Intersection I** ^**1**^	DEGs from the four contrasts in dataset 1: CT1; CT2; CT3; CT4	DEGs from contrasts including EE2-treated fish versus male and female control fish: MT-MC D7 to D111; MT-FC D7 to D111.
**Intersection II** ^**2**^	DEGs from the four contrasts in dataset 1: CT1; CT2; CT3; CT4	DEGs from contrasts including EE2-treated fish versus male control fish: MT-MC D7 to D111.
**Intersection III** ^**3**^	CT1; CT2. *These two contrasts were previously correlated with gonad intersex feature* ^*4*^.	MT-MC D7; MT-MC D16; MT-MC D30. *These contrasts cover early developmental stages corresponding to differentiating gonads*, *with first morphological differences appearing between male and female gonads*.
**Final intersection** ^**5**^	DEG selected in intersection III : (i) common to pathway/Ontologies selected with intersections I and II (ii) selected by vizualization techniques (Volcanoplots and kinetic profiles)	DEG selected in intersection III : (i) common to pathway/Ontologies selected with intersections I and II (ii) selected by vizualization techniques (Volcanoplots and kinetic profiles)

^**1**^ Larger gene list retrieving genes common to both datasets. This list was used for pathway analysis. We postulated that it represents mechanistic signatures of EE2 treatment on rainbow trout juveniles.

^**2**^ Shorter gene list retrieving genes common to both datasets. This list was used for ontology analysis. We postulated that it represents mechanistic signatures of EE2 treatment on rainbow trout juveniles.

^**3**^ Shortest gene list retrieving genes common to both datasets. We postulated that it focuses on genes potentially involved in early responses to EE2 treatment. This third group was selected based on union intersections, which represents DEGs common to at least one condition in dataset 1 and one condition in dataset 2 (for example DEG in CT1 and MT-MC D7).

^4^ [25]

^**5**^ Limited number of genes considered true positives after all filtering steps of the analysis workflow. We postulated that these genes are potentially implicated in male-to-female transdifferentiation processes. Gene expression profiles of this final gene list were compared with gene expression between normal (control) male and female juvenile rainbow trout.

### 2.1. Dataset 1 [24]

In this experiment, all-male rainbow trout (*Oncorhynchus mykiss*) fry (INRA experimental fishfarm, Monts d'Arrée-PEIMA, Sizun, France) were exposed from the onset of first feeding [Day 0 = D0 at 60 days post-fertilization (dpf)] to 136 dpf to 5 nominal concentrations of 17α-ethynylestradiol (purity ≥ 98%, Sigma-Aldrich, Germany): 0 (solvent control), 0.01 μg/L, 0.1 μg/L, 1 μg/L and 10 μg/L, with 3 tanks per condition. The actual EE2 concentrations were measured in each tank at 6 time points using the Quantitative Ethynylestradiol Enzyme Immunoassay (EIA) Kit (Marloie, Belgium) according to the manufacturer’s instructions. Mean concentrations of EE2 ± SD were 0.08 ± 0.06 μg/l; 1.62 ± 1.74 μg/l and 9.88 ± 5.06 μg/l. The 0.01 μg/l EE2 concentration was under the detection limit (set at 0.02 μg/l). At the end of the exposure, the gonads were collected from all fish, immediately frozen in liquid nitrogen and stored at -80°C until RNA extraction. The gonads from ten fish were pooled to reach enough material for further analyses. Gene expression measurement was performed on 6 replicates per condition (2 pools of gonads per tank) on a one-color 8x60K oligonucleotide array (Agilent Technologies) designed by the INRA-LPGP microarray platform (Beaulieu Campus, Rennes, France). Expression profiles obtained from the DNA microarray dataset were validated with the measurement of several genes by real-time reverse transcription-polymerase chain reaction (RT-PCR) as described previously [25].

### 2.2. Dataset 2 [26]

In a previous experiment (Guiguen *et al*., unpublished data), transcript expression was recorded over a time-course analysis on gonads sampled following feminization of genetically all-males population (M-control) with estrogens (M-EE2) or masculinization of genetically all-females population (F-control) with either androgen (F-11ß) or an anti-aromatase (F-ATD). The present analysis is limited to estrogen-treated fish. Feminizing treatment of all-male populations (PEIMA, Sizun, France) with 17α-ethynylestradiol (EE2, 20 mg/kg of food, Sigma, St Louis, MO, USA) was applied for 2 months starting from the first feeding. In each group, 20 to 100 gonads were sampled and pooled in duplicate corresponding to the various stages of development: onset of the free swimming period after complete yolk resorption [Day 0 = D0 at 55 dpf], D0+7 days (D7), D16, D30, D63, D91, and D111. Gonads were immediately frozen in liquid nitrogen and stored at -80°C until RNA extraction. Gene expression analyses were carried out using home-made one-color Nylon DNA microarrays obtained as previously described [6] and containing 9,023 trout cDNA clones (TROUT-AGENAE). Expression profiles obtained from the DNA microarray dataset were validated with the measurement of several genes by real-time reverse transcription-polymerase chain reaction (RT-PCR).

### 2.3. Microarray analysis workflow

The initial microarray analysis workflow [24] was adapted to handle another type of microarray platform (dataset 2) and to fit with the meta-analysis scheme and work hypothesis. Briefly, this workflow includes (i) the analysis of pre-treated data by (ii) ANOVA analysis followed by Scheffe’s post hoc pairwise comparisons. Thereafter, (iii) biologically relevant sets of contrasts and gene list intersections between the two datasets were selected at different levels of stringency. The remaining gene lists, supposed to be enriched in true positive DEGs, were (iv) submitted to over-representation analysis methods (ORA). (v) A set of most relevant DEG was selected using volcanoplots, membership of enriched pathways and/or GOterms and kinetic profiles in both analyses. (vi) Those genes were compared with normal expressions profiles between control rainbow trout male and female developing fry.

### 2.4. Statistical analysis

Data analyses were performed using the R statistical software version 2.15.3 available on the R-Project website (http://cran.r-project.org) and a set of packages available in the Bioconductor repository (http://www.bioconductor.org). This methodology used for the first dataset [24] was also applied to re-analyse the dataset 2. Therefore, brief descriptions and specificities related to this dataset analysis are provided below. See supporting information for detailed scripts.

#### 2.4.1. Pre-processing and normalisation procedures

The dataset was configured in numeric matrix. The expression values were first submitted to a quantile-quantile normalisation using the normalize.quantile function from the preprocessCore package (S1 Dataset).

#### 2.4.2. ANOVA

A two-way ANOVA was performed, with the time-course (D0 to D 111) and sex as criteria, using the lmFit function of package Limma, the only statistical method able to perform ANOVA with more than one factor level (here the 7 developmental stages tested) on microarray data. Its predictive power has been well ranged in our previous benchmark of method performances [27].

#### 2.4.3. Post-hoc comparisons

A post-hoc evaluation was performed using Scheffe’s method with the makeContrasts() and eBayes() functions provided in the Limma package (88 contrasts comparing all experimental conditions). The results were summarized using decideTests() and summary() (number of significant genes positively or negatively regulated for each evaluated contrast). The Benjamini-Hochberg procedure was used to adjust the p-values (correction for multiple testing) [28]. Considering the very large number of genes per contrast group, we later focused the analysis of dataset 1 on four groups that were the most relevant with regards to the biological context, comparing reference samples with samples treated with EE2 at different concentrations. The contrast labels were defined as control vs. different EE2 concentrations—[EE2]: (i) CT1 = Control vs. [0.01 μg/L]; (ii) CT2 = Control vs. [0.1 μg/L]; (iii) CT3 = Control vs. [1 μg/L] and (iv) CT4 = Control vs. [10 μg/L].

For the 2^nd^ dataset, a differential gene expression analysis was performed using the two-way ANOVA, considering the developmental stages (D0 to D111) and sex (male and female) as criteria. Eighty-eight contrasts were performed comparing all the experimental conditions. We postulated that gene expression comparisons make sense between fish at the same developmental stage. To this end, we defined 19 physiologically relevant contrasts as follows: (i) we selected the 7 male-EE2 (= male test = MT) versus male-control (MC) contrasts at the same sampling time (MT-MC D7, MT-MC D16, MT-MC D30, MT-MC D63, MT-MC D91, MT-MC D111); (ii) the 7 male-EE2 versus female-control (FC) contrasts at the same sampling times (MT-FC D7, MT-FC D16, MT-FC D30, MT-FC D63, MT-FC D91, MT-FC D111), and (iii) the 8 male-control (MC) versus female-control (FC) contrasts at the same sampling times (MC-FC D0, MC-FC D7, MC-FC D16, MC-FC D30, MC-FC D63, MC-FC D91, MC-FC D111). For each contrast, R software topTable() function was used to extract top-ranked genes and relevant statistics (logFC, Average Expression, t statistic, p-value, adjusted p-value, B statistic).

#### 2.4.4. Meta-analysis

In theory, meta-analysis of microarray data consists of analyzing several related datasets at once [22]. The statistical analysis is then made on merged datasets. In our analysis, this scheme was impossible as the platforms used were heterogeneous. Here, the meta-analysis consists of retrieving DEGs common to both analyses. The use of intersections between top gene lists has proven to be effective in filtering relevant genes [19,22,24]. Three intersections (Intersection I, II and III) were defined for further analyses (Table 1).

Intersection I has been proposed to retrieve genes common between the two DEGs lists from the two datasets. We postulated that this represents mechanistic signatures of EE2 treatment on rainbow trout male juveniles. This group therefore contained the intersection between all DEGs in the four contrasts in dataset 1 (CT1 to CT4) and all DEGs in the twelve contrasts including EE2-treated fish in dataset 2 (MT-MC D7 to D111, and MT-FC D7 to D111). This group contained 1,535 DEGs and was further used in the pathway overrepresentation analysis (see result section 3.2.1.).

To further focus on genes differentially expressed in male treated fish, a smaller group of DEGs (Intersection II) was obtained at the intersection between all DEGs in the four contrasts in dataset 1 (CT1 to CT4) and all DEGs in the six contrasts including EE2-treated fish versus male control fish in dataset 2 (MTMC D7 to D111). This group contained 438 DEGs and was further used in the ontology overrepresentation analysis (see result section 3.2.2.).

To further investigate the trans-differentiation process, we aimed to focus on genes potentially involved in the early response to EE2 treatment (Intersection III). The statistical and bioinformatics approaches applied here were aimed at retrieving true positives among contaminated large gene lists. For this, we focused on the DEGs related to the lower doses used in dataset 1 (corresponding to contrasts CT1 and CT2). We have reported previously that these conditions are correlated with gonad intersex feature development in rainbow trout male juveniles exposed to EE2 [24]. In dataset 2, the interesting contrasts were those at the early developmental stages, 7, 16 and 30 days after treatment. In developing rainbow trout, this period corresponds to differentiating gonads, with the first morphological differences appearing between male and female gonads. Day 12 corresponds to the occurrence of oocyte meiosis and D27 to the beginning of ovarian lamellar structure development in females. Gametogenesis starts from 60 dpf [29]. The third group was composed of the union intersection between contrasts CT1 and CT2 in dataset 1 and MT-MC D7, MT-MC D16 and MT-MC D30 in dataset 2. The union intersection included DEGs common to at least one condition in dataset 1 and one condition in dataset 2 (for example DEG in CT1 and MT-MC D7). This retrieved DEGs that could be false negatives in other contrasts considered. To further filter these DEGs, (i) we confronted this list to the pathways and ontologies retrieved in previous steps. Thereafter, (ii) we used visualization techniques. First (a), we submitted them to a Volcanoplot analysis, then (b) we looked at their kinetic profiles in both analyses. Finally, the intersection of gene lists retrieved by each approach was selected. (iii) The final gene list was then compared with DEGs between normal male and female differentiation.

#### 2.4.5. Annotations

Considering that the recently available genome of rainbow trout has not yet a complete and accurate functional annotation, no consensus exist for the annotation of the Agilent 60K array and for the AGENAE_TroutGeneric2_9216 platforms. Bioinformatics tools have different requirements concerning the gene IDs to enter in the program, but commonly they need homogenous identifiers and no redundancy. In addition, the annotation of oligonucleotides improves daily and can change substantially within several months. Therefore, it is important to use the most accurate annotation at the time of analysis. To be able to use DAVID tool (see below), we used ensembl gene ID for *Danio rerio* (ENSDARG), the only fish species with pathways available on Kegg [30]. For EASE, as no entry exists for ensembl gene ID, Gene Symbol identifiers were used, because the most accurate. We have updated the latest version of the AGENAE program (January 2013) (Analysis of Breeding Animals’ Genome; http://www.agenae.fr/programme_agenae/) for these two annotations, using UniProt (http://www.uniprot.org) or e!Ensembl (http://www.ensembl.org) databases.

#### 2.4.6. Visualizations

Several bioinformatics tools were used to gain insights of the dataset, at different levels of organization (gene expression, pathways, ontologies) depending on the analysis stringency of the group of DEG tested. The “Intersection I” group of DEG (see results) was submitted to the web tool DAVID (Database for annotation, Visualization and Integrated Discovery; http://david.abcc.ncifcrf.gov/home.jsp) [31,32], to retrieve pathway maps from KEGG following the method applied by Pierre et al, 2010 [19], and with the genes on the 60K rainbow trout Agilent array set up as the ‘background population’. The “Intersection II” group was submitted to the EASE software (version 2.0)(Expression Analysis Systematic Explorer) available on the DAVID website [33]. EASE is a desktop version of DAVID allowing more freedom toward the identifiers entering the program, which suits our heterogeneous Swissprot annotation (several species orthologs). Gene symbol was selected in the Input Genes setting. Then we choose the “Find over represented gene categories” function. In the “Population file” window we enter the entire array annotation without redundancy. Then we set the “Run Basic Analysis” function. Finally, Volcanoplots were made to visualize the DEG of interests using the plot function in the R software. A volcanoplot is a graph sorting the results by p-value (Y-axis),-log fold change (X-axis) and over/under-expression. In such graphs, the most interesting genes are usually located in the upper left and right corner of the plot, depicting genes with low p-values and high fold changes.

## Results

### 3.1. ANOVA and Scheffe’s contrasts

**Fig 1:**
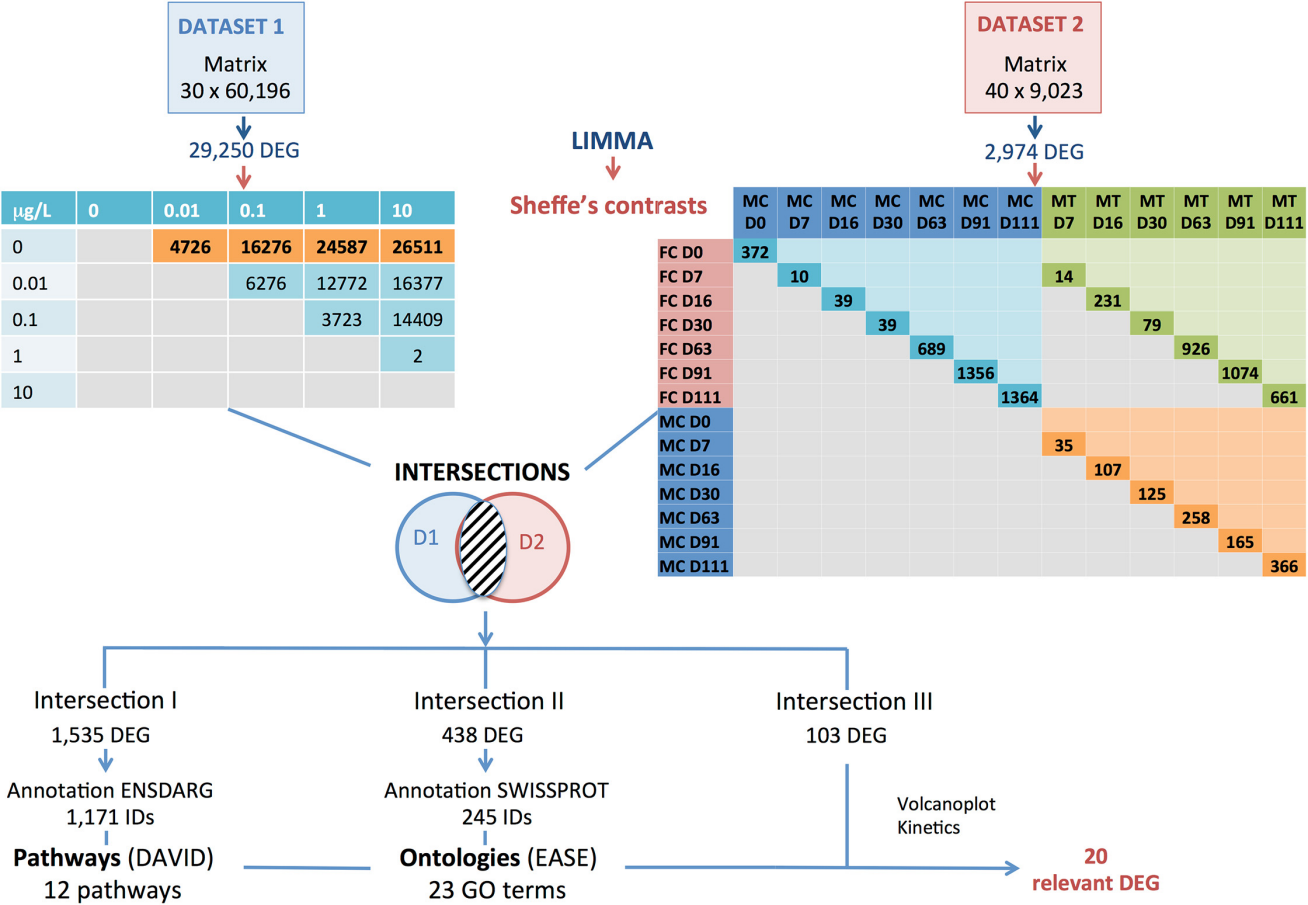
Flowchart of the workflow of the microarray meta-analysis methodology and associated results. This flowchart summarizes the bioinformatics workflow used for the meta-analysis of two datasets studying sex reversal following exposure to EE2 in juvenile rainbow trout. Both datasets (dataset 1 and dataset 2, pre-treated data) were subjected to ANOVA analyses followed by Scheffe’s post hoc pairwise comparisons. Thereafter, biologically relevant sets of contrasts (coloured and in bold in the flowchart) were selected (differentially expressed genes (DEGs) between control and EE2-treated fish in dataset 1; DEGs between control and EE2-treated fish at the same developmental stage in dataset 2). The meta-analysis consisted of retrieving the intersection of the top DEGs lists generated for both datasets, performed at different levels of stringency. This allowed creating different groups of DEGs (**Intersections I, II and III**) for ORA analyses downstream, given that pathway or Ontologies research have different constraint concerning the group of genes to enter in the program. It is worth noting that each group of DEGs has different biological meaning described further in the text in the results section. **Intersection I** (low stringency) contained the intersection between all DEGs in the four contrasts in dataset 1 (CT1 to CT4) and all DEGs in the twelve contrasts including EE2-treated fish in dataset 2 (MT-MC D7 to D111, and MT-FC D7 to D111). This group contained 1,535 DEGs (1,171 DEGs having a functional annotation with *Danio rerio* Ensembl! identifiers-ENSARG) and was further used in the pathway overrepresentation analysis. A smaller group of DEGs (**Intersection II**) was obtained by retrieving the intersection between all DEGs in the four contrasts in dataset 1 (CT1 to CT4) and all DEGs in the six contrasts including EE2-treated fish versus male control fish in dataset 2 (MTMC D7 to D111). This group contained 438 DEGs (245 DEGs having a functional annotation with Swissprot identifiers) and was further used in the ontology overrepresentation analysis. The third group (**Intersection III**–highest stringency– 103 DEGs) was composed of the union intersection between contrasts CT1 and CT2 in dataset 1 and MT-MC D7, MT-MC D16 and MT-MC D30 in dataset 2. From this last list of DEGs, a set of most relevant DEGs was selected using volcanoplots, membership of enriched pathways and/or GOterms and kinetic profiles in both analyses. The 20 remaining genes, considered as potentially implicated in the transdifferentiation processes, were selected for further analyses.

Fig 1 summarizes the microarray meta-analysis workflow and shows an overview of the associated results. The tables of contrasts include redundant genes.

The results for the 10 contrasts, presented by number of differentially expressed genes without redundancy but before annotation, are shown in Fig 1 (left-hand table). The cut-off for DEG inference was set to 0.05 for the adjusted p-values (Benjamini-Hochberg). The four contrasts previously defined in section 2.4.3 for the 1^st^ dataset represent 12,742 non-redundant DEGs (sum of the orange cells in the left-hand table in Fig 1, with subtraction of the redundant genes, namely genes present in several contrasts). For the 2nd dataset, the contrasts represent 737 (in orange), 1,878 (in green) and 2,305 non-redundant DEGs (in blue) respectively (Fig 1).

### 3.2. Meta-analysis on selected of groups of DEGs (Intersection I, II and III)

#### 3.2.1. Intersection I: pathway over-representation analysis

The list of Ensembl gene IDs for *Danio rerio* corresponding to the “Intersection I” DEG group was used for pathway over-representation analysis. Of the 1,535 DEGs in intersection I, 1,171 were accurately annotated with an ENSDARG ID and recognized by the DAVID web tool. The background was made up of 16,977 ensembl IDs (corresponding to the total number of identifiers on the Agilent 60K platform). A total of 79 pathways containing at least 2 genes were identified from the remaining 1,171 genes (S1 Results). Among these pathways, 12 were detected with a significant enrichment in the list of DEGs submitted, with an EASE score below the threshold of 0.05 (Table 2).

**Table 2:** List of significantly enriched pathways. The list of significantly enriched pathways (P-Value/EASE score < 0.05) was obtained by using the DAVID web tool for the Intersection I group in our meta-analysis (1,535 gene IDs restricted to a set of 1,171 well annotated differentially expressed genes recognized by ENSDARG identifiers in DAVID). This group represents the genes differentially expressed following steroid modulation of immature gonads in rainbow trout by EE2, under several types of treatment. The Count column gives the number of genes of our genelist belonging to the pathway. The EASE-Score column represents the P-Value calculated by DAVID. The Benjamini column represents the Benjamini-corrected P-values (correction for multiple testing).

Term	Count	EASE-score	Benjamini
Cell cycle	34	1.1^E^-6	1.3^E^-4
DNA replication	15	2.9^E^-5	1.8^E^-3
Oxidative phosphorylation	29	6^E^-5	2.5^E^-3
Pyrimidine metabolism	20	3.7^E^-3	1.1^E^-1
Nucleotide excision repair	12	8.2^E^-3	1.8^E^-1
Mismatch repair	8	1.1^E^-2	2^E^-1
Fatty acid elongation in mitochondria	5	1.5^E^-2	2.4^E^-1
Ribosome	18	1.6^E^-2	2.2^E^-1
PPAR signaling pathway	12	3.4^E^-2	3.8^E^-1
RNA degradation	12	4.4^E^-2	4.2^E^-1
Progesterone-mediated oocyte maturation	16	4.7^E^-2	4.2^E^-1
Valine, leucine and isoleucine degradation	10	4.8^E^-2	3.9^E^-1

**Fig 2:**
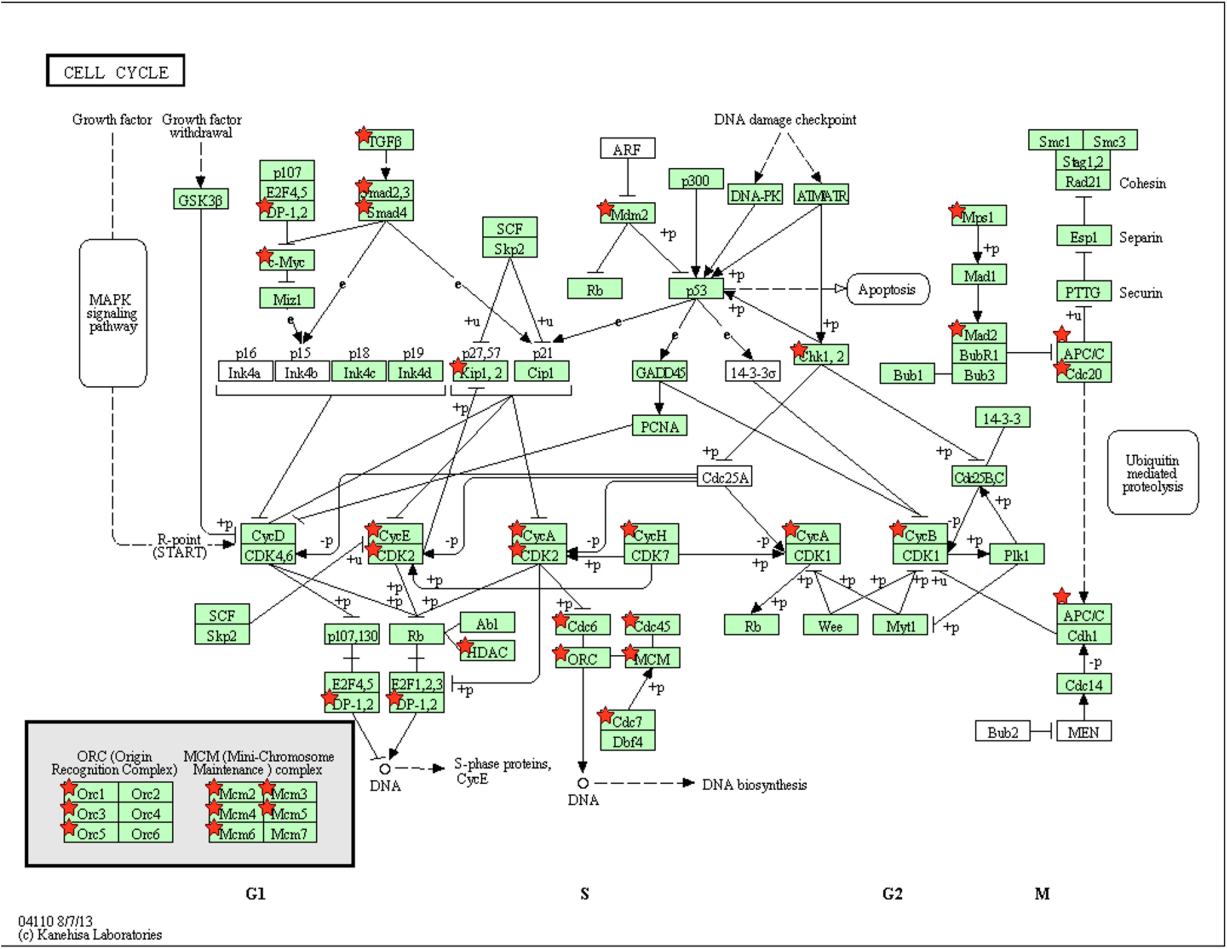
Cell cycle. In our study, the Cell Cycle is an example of a significantly enriched pathway resulting from the DAVID analysis. Red stars indicate genes belonging to the list of DEG submitted to the program. The background was made up of 16,977 Ensembl IDs (corresponding to the total number of identifiers on the Agilent 60K platform).

Only three pathways (Cell cycle, DNA replication and oxidative phosphorylation) could be detected when the threshold was set to adjusted p-values, after the highly conservative Benjamini multiple testing corrections (see Table 2). Most of these enriched pathways are involved in Genetic Information Processing, including replication and repair (DNA replication, Nucleotide excision repair, Mismatch repair) but also translation (Ribosome) and degradation (RNA degradation). Several pathways belonged to Metabolism, with energy (Oxidative phosphorylation), nucleotide (pyrimidine metabolism), lipid (fatty acid elongation) and amino acid (valine, leucine and isoleucine degradation) groups represented. Of particular interest were those pathways related to cellular processes: the “Cell cycle” was the most significantly enriched set of genes, with the highest number of highlighted genes (34), but the “Endocrine system” was also identified with PPAR signalling pathway and Progesterone mediated oocyte maturation. Gene enrichment is illustrated with red stars corresponding to the DEGs retrieved in our analysis (Fig 2).

#### 3.2.2. Intersection II: ontology over-representation analysis

The list of Ensembl gene IDs for *Danio rerio* corresponding to the “Intersection II” DEG group was used for ontology over-representation analysis. Of the 438 DEGs of this intersection, 245 could be accurately annotated. Interestingly, 58 terms were significantly enriched (EASE score less than 0.05). Only GO Terms with highly significant EASE scores (p-values less than 0.01) were considered. Table 3 provides a summary of the results obtained. An exhaustive presentation of the relationships between all genes and each category is provided in S2 Results. Genes that belonged to the “Intersection II” set could be summarized in four broad terms with high significance: (i) the most significant terms belonged to “Reproduction” (more precisely, sexual reproduction and gametogenesis). Interestingly, several genes were related to male gamete maturation and spermatogenesis; (ii) several enriched terms were related to the cellular process (cell cycle, cell proliferation,…) and more precisely to mitosis (mitotic cell cycle, M phase, S phase, …); (iii) several terms belonged to the metabolic process (oxidoreductase activity, DNA metabolism, …); (iv) DNA replication was overrepresented.

**Table 3:** List of significantly enriched GO terms. This table lists the significant GO terms (P-Value/EASE score < 0.01) retrieved by the EASE software for the Intersection II group in this analysis (245 well annotated DEGs recognized by Gene Symbol identifiers in EASE among 438 DEGs IDs). This group represents the DEGs following a steroid modulation of immature gonads in rainbow trout by EE2, under several types of treatment. The EASE-Score column represents the P-Value calculated by DAVID.

GO ID	Term	EASE score (P-Value)
GO:0019953	Sexual reproduction	3.2^E^-07
GO:0000003	Reproduction	3.2^E^-07
GO:0007276	Gametogenesis	1.7^E^-06
GO:0008283	Cell proliferation	5.9^E^-05
GO:0007049	Cell cycle	7.2 ^E^-05
GO:0000278	Mitotic cell cycle	1.4^E^-04
GO:0000074	Regulation of cell cycle	1.0^E^-03
GO:0006261	DNA dependent DNA replication	1.0^E^-03
GO:0005743	Mitochondrial inner membrane	1.0^E^-03
GO:0009117	Nucleotide metabolism	1.3^E^-03
GO:0019866	Inner membrane	2.0^E^-03
GO:0005737	Cytoplasm	2.3^E^-03
GO:0000279	M phase	3.1^E^-03
GO:0005694	Chromosome	3.2^E^-03
GO:0016491	Oxidoreductase activity	3.5^E^-03
GO:0005740	Mitochondrial membrane	3.9^E^-03
GO:0006220	Pyrimidine nucleotide metabolism	4.5^E^-03
GO:0006260	DNA replication	5.1^E^-03
GO:0006259	DNA metabolism	5.4^E^-03
GO:0000084	S phase of mitotic cell cycle	5.8^E^-03
GO:0048232	Male gamete generation	8.4^E^-03
GO:0007283	Spermatogenesis	8.4^E^-03
GO:0019201	Nucleobase, nucleoside, nucleotide kinase activity	9.1^E^-03

#### 3.2.3. Intersection III: membership of pathway/ontologies, volcanoplots and kinetics

Intersection III group contained 103 DEGs. They were compared with genes belonging to enriched Pathways and/or GO Terms highlighted earlier. Among the 103 DEGs, 44 belong to at least one enriched pathway and/or GO Term. Several of these listed genes appeared to be ubiquitous. For example, *RFC3* can be found in the DNA replication pathway as well as GO Terms related to the Cell cycle, Cell proliferation, and the Chromosome. *Ropn1* appeared in Spermatogenesis, Cell proliferation and the mitochondrial membrane.

Two visualization techniques were then used to select genes of interest. First we selected the most significant genes in terms of Fold change and P-Value by Volcanoplot visualization over all the contrasts considered. Second, the kinetic profiles of remaining genes were visualized in both datasets.

**Fig 3:**
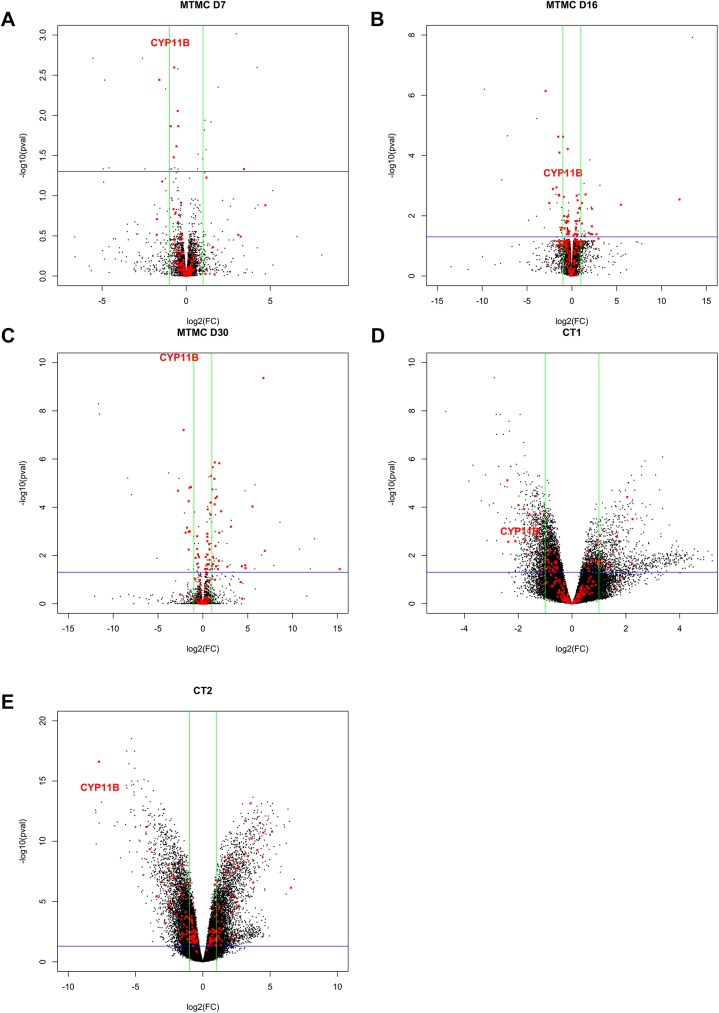
A-E: Volcano plots. These graphs represent the 103 DEGs belonging to the ‘Intersection 3‘ group of DEGs selected through the intersection between low doses (contrasts CT1 and CT2) in the Dataset 1 and early stages (contrasts MT-MC D7, D16 and D30) in dataset 2. For sake of clarity, red points represent DEGs. As an example, CYP11B is represented by its label. The green bars represent the log _2_(+/-2) fold change and the blue bar represents a p-value threshold of 0.05. (A) Selected DEGs in contrast MTMC D7 in dataset 2. (B) Selected DEGs in contrast MTMC D16 in dataset 2. (C) Selected DEGs in contrast MTMC D30 in dataset 2. (D) Selected DEGs in contrast CT1 in dataset 1. (E) Selected DEGs in Contrast CT2 in dataset 1.

Volcanoplots are presented in Fig 3. These graphs show the comparison between the five contrasts considered for the 103 DEGs of the Intersection III group. DEGs were selected from these graphs as follows (i) Location in the right or left corners of the graphs (ii) Membership of at least two contrasts between dataset 1 and dataset 2, and (iii) Consistency of fold change type (positive or negative) between both involved contrasts. In total, 60 DEGs were selected by this approach. Among this list, 23 DEGs belonged to enriched Pathways/GO Terms. For the sake of clarity, only cyp11b is represented on graphs, which was interestingly found at a very similar position in all the contrasts considered (i.e. left corner of the graph).

**Fig 4:**
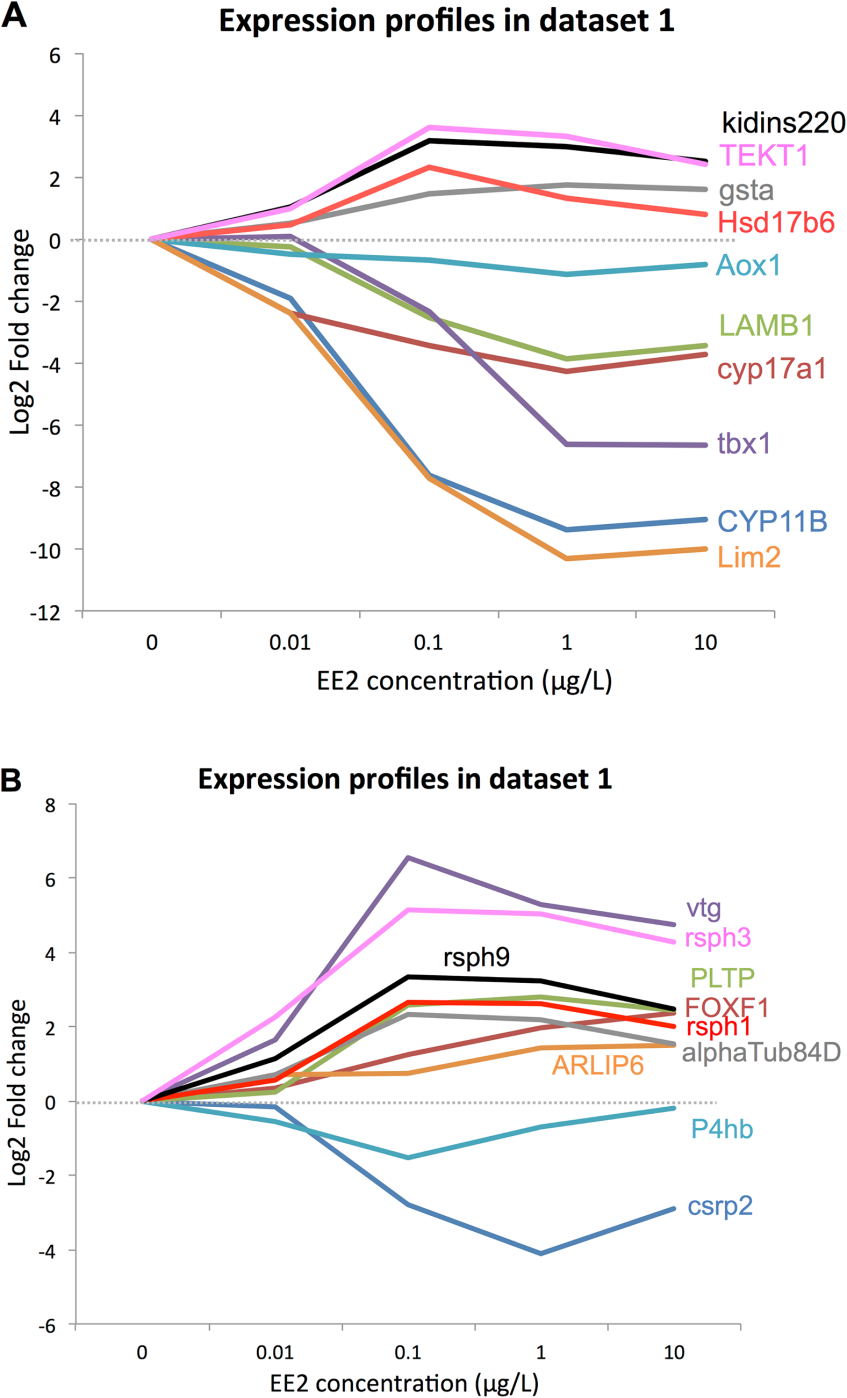
A-B: Selected genes kinetics profiles over the several concentrations tested in dataset 1. These graphs show the profile of the 20 DEGs retrieved by the analysis over the 4 concentrations tested in dataset 1. X-axis: EE2 concentrations (0 = control). Y-axis: fold change of the different EE2 concentrations vs CTL. **A:** expressions of the 10 genes also differentially expressed between female and male control fish. **B:** expressions of the 10 genes specific to EE2 treatment.

**Fig 5:**
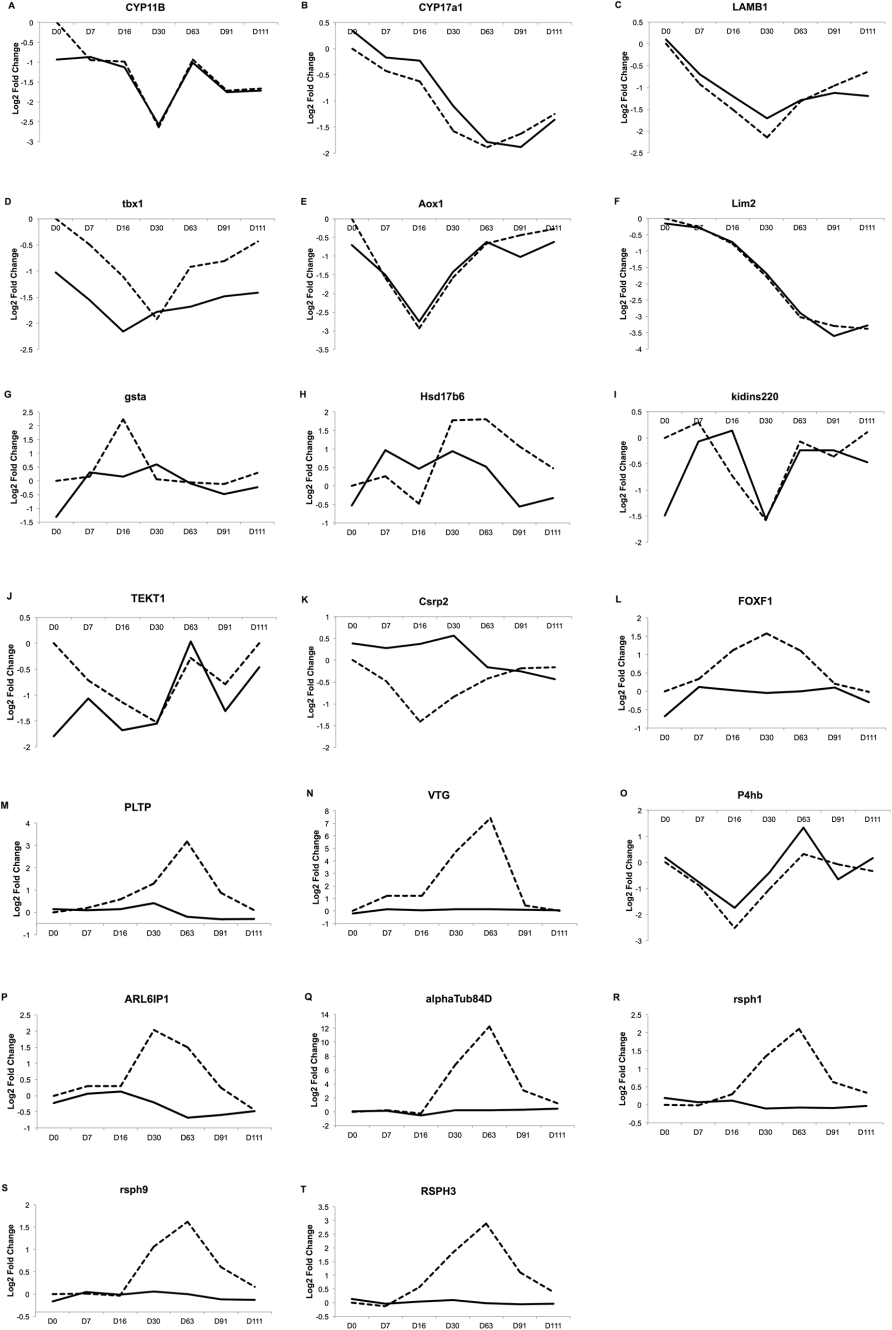
A-T: Selected genes kinetics profiles over the several sampling time in dataset 2. These graphs show the profile of the 20 DEGs retrieved by the meta-analysis in dataset 2. Moreover, a comparison between gene expression between male exposed to EE2 versus male control and the expression in a control female versus male control is made. Y-axis: fold change of gene expression between males exposed to 20mg/kg of food of ethynylestradiol (dotted lines) or control females (solid lines) at each developmental stage versus male control at the same developmental stage (contrasts MT-MC D7 to D111 and FC-MC D0 to D111, Fig 1). X-axis: sampling time. Day 0 = D0 at 55 dpf, Day 7 = D0+7 days, D16, D30, D63, D91, and D111.

The next step was to look at the kinetics profiles of the 60 DEGs selected by the Volcanoplot analysis from the two whole datasets considered (CT1 to CT4 in dataset 1 and MTMC D7 to D111 in dataset 2). The kinetics profiles for the 20 genes in dataset 1 and dataset 2 are given in Fig 4A and 4B and Fig 5A–5T, respectively. Selection was based on deleting genes whose maximum expression was under 1.5 in overexpressed genes or over -1.5 in underexpressed genes. This retrieved 42 DEGs in dataset 1 and 22 DEGs in dataset 2. Through the identification of DEGs common to these two lists, we found 20 DEGs of particular interest (Table 4). Among these genes, 7 belonged to significantly enriched Pathways/GO Terms. It is worth noting that, among the 20 DEGs selected, there were several exceptions. *TEKT1* and *Kindiss* have opposing fold changes, as they are overexpressed in dataset 1 and underexpressed in dataset 2. Moreover, *Aox1* in dataset 1 and *Crsp2* in dataset 2 have a maximum log2 (Fold Change) expression over -1.5. However these genes were selected in Volcanoplots and displayed interesting expression patterns, with a peak at low concentrations in dataset 1 and/or at early time points in dataset 2. They were then kept as interesting genes.

**Table 4:** Description of the 20 genes selected as physiologically relevant in the gonad transdifferentiation process. Fold change (FC) and pvalues (Pval) are given for the five contrasts considered (CT1 and CT2 in dataset 1; MT-MC D7, D16 and D30 in dataset 2), upon which the genes were selected. Figures in bold represents significant values. The membership of genes to significantly enriched pathway and/or GO Terms is indicated.

Gene symbol	Description	FC CT1	Pval CT1	FC CT2	Pval CT2	FC MTMC D7	Pval MTMC D7	FC MTMC D16	Pval MTMC D16	FC MTMC D30	Pval MTMC D30	Pathway	GO Term
**CYP11B**	Cytochrome P450 11B	**-3.8**	**9.8E-04**	**-200.3**	**3.3E-15**	**-1.93**	**1.2E-03**	**-2.0**	**3.7E-04**	**-6.2**	**6.0E-11**	**-**	**-**
**Cyp17a1**	Steroid 17-alpha-hydroxylase/17,20 lyase	**-5.2**	**2.7E-03**	**-10.8**	**3.7E-06**	-1.34	5.5E-01	-1.5	9.1E-02	**-3.0**	**5.7E-05**	-	Oxidoreductase activity
**Csrp2**	Cysteine and glycine-rich protein 2	**-1.1**	**1.5E-02**	**-6.9**	**6.7E-04**	-1.39	5.6E-01	**-2.7**	**2.1E-03**	-1.8	9.7E-02	-	Cell proliferation
**Lamb1**	Laminin subunit beta-1	-1.2	6.5E-01	**-5.8**	**8.9E-09**	**-1.89**	**1.4E-02**	**-2.9**	**2.4E-05**	**-4.4**	**6.3E-08**	-	-
**Tbx1**	T-box transcription factor 1	1.1	9.1E-01	**-5.2**	**3.7E-07**	-1.41	7.1E-01	-2.2	7.1E-02	**-3.8**	**1.1E-03**	-	Cytoplasm
**Aox1**	Aldehyde oxidase	-1.4	2.8E-01	**-1.6**	**4.8E-02**	-3.03	3.6E-03	**-7.6**	**7.2E-07**	**-3.0**	**1.1E-03**	-	Oxidoreductase activity
**FOXF1**	Forkhead box protein F1	1.3	5.4E-01	**2.4**	**2.0E-03**	1.27	6.6E-01	**2.2**	**3.8E-03**	**3.0**	**3.6E-05**	-	-
**PLTP**	Phospholipid transfer protein	1.2	7.9E-01	**6.0**	**2.8E-05**	1.14	7.7E-01	**1.5**	**4.6E-02**	**2.4**	**6.6E-06**	PPAR signaling	-
**Vtg1**	Vitellogenin	3.2	2.6E-01	**93.4**	**7.1E-07**	2.32	8.3E-01	2.3	5.7E-01	**27.2**	**3.4E-02**	-	-
**Lim2**	Lens fiber membrane intrinsic protein	**-5.3**	**7.6E-06**	**-211.8**	**2.5E-17**	-1.20	8.0E-01	-1.7	7.1E-02	**-3.4**	**7.1E-04**	-	-
**P4hb**	Protein disulfide-isomerase	-1.5	5.1E-01	**-2.9**	**8.8E-03**	-1.88	5.6E-01	**-5.7**	3.8E-03	-2.0	5.3E-01	-	-
**ARL6IP1**	ADP-ribosylation factor-like protein 6-interacting protein 1	1.6	1.7E-02	**1.7**	**2.2E-03**	1.23	8.4E-01	1.2	5.7E-01	**4.1**	**1.5E-04**	-	-
**gsta**	Glutathione S-transferase A	1.4	2.6E-01	**2.7**	**6.2E-05**	1.12	9.5E-01	**4.7**	2.2E-02	1.0	1.0E+00	-	-
**HSD17b6**	17-beta-hydroxysteroid dehydrogenase type 6	1.4	2.6E-01	**5.1**	**1.3E-08**	1.20	9.2E-01	-1.4	5.0E-01	**3.4**	**1.7E-02**	-	-
**alphaTub84D**	Tubulin alpha chain	**1.6**	**2.7E-02**	**5.1**	**1.7E-08**	1.15	9.5E-01	-1.2	7.5E-01	**107.4**	**4.4E-10**	-	-
**rsph1**	Radial spoke head 1 homolog	1.5	2.1E-01	**6.2**	**1.4E-08**	-1.01	9.9E-01	1.2	1.8E-01	**2.5**	**1.4E-06**	-	-
**kidins220**	Kinase D-interacting substrate of 220 kDa	**2.1**	**1.5E-03**	**8.9**	**1.9E-12**	1.23	8.0E-01	-1.7	1.0E-01	**-3.0**	**5.7E-03**	-	-
**rsph9**	Radial spoke head protein 9	**2.2**	**2.2E-02**	**10.2**	**1.7E-09**	1.01	1.0E+00	-1.0	8.8E-01	**2.1**	**1.8E-05**	-	Cytoplasm
**Tekt1**	Tektin-1	**2.0**	**2.4E-02**	**12.1**	**5.8E-11**	-1.64	3.5E-01	-2.2	2.5E-02	**-2.9**	**1.0E-03**	-	-
**RSPH3**	Radial spoke head protein 3	**4.7**	**3.1E-04**	**35.4**	**1.6E-11**	-1.09	9.1E-01	1.5	8.5E-02	**3.6**	**1.5E-06**	-	Cytoplasm

In order to focus on relevant genes potentially related to early transdifferentiation processes, the final step was to compare the final list of 20 DEGs to differential gene expression existing between female and male control fish. For this purpose, we compared the list of 20 DEGs with all the DEGs in the contrasts in dataset 2 comparing DEGs between control females versus control males in the developmental stages considered (MC-FC D7, D16 and D30, Table of contrasts in Fig 1 in blue). Ten out of the 20 genes were also differentially expressed between control females and control males (Fig 5A–5T). Interestingly, the 10 genes also differentially expressed in females (Fig 5A–5J) are mostly underexpressed genes and very similar expression profiles between EE2-treated fish and control females are observed at all developmental stages considered. The 10 remaining genes (Fig 5K–5T), considered specific to EE2 treatment, were mostly overexpressed genes, with peak expression at D30. They shared very similar expression patterns.

## Discussion

### 4.1. Differentially expressed genes and pathways

Both dataset analyses generated a high number of differentially expressed genes (DEG) following EE2 treatment, with nearly half of the array content displaying differential expression patterns in dataset 1 (12,742 non-redundant genes), and one third in dataset 2 (2,974 non-redundant genes). Since EE2 treatment is able to induce complete sex reversal of fish gonads, it is not surprising that this impact was detected at the transcriptional level. A similar magnitude of gene expression disruption was also observed in female fish masculinized by androgen 11β-hydroxyandrostenedione [6]. This highlights the major role of steroid hormones in triggering the gonad transcriptome, and their potency. Interestingly, 1,535 DEGs were found to be common to both analyses. These genes are therefore of particular interest considering their implication in the response of juvenile rainbow trout to EE2 treatment. The bioinformatics workflow applied in this study was previously shown to be effective in retrieving pathways in order to focus on potential novel genes associated with cancer metastasis and hypoxia in humans [19,22,23], and to identify potential biomarkers of intersex in rainbow trout [24].

Intersections between groups of DEGs highlight genes implicated in the response of juvenile rainbow trout to exogenous EE2 treatment. To focus on relevant genes related to the male-to-female transdifferentiation process, we further filter on phenotypic anchoring, which consists of encompassing different levels of biological organization [11]. We choose the CT1 and CT2 contrasts as the most relevant in dataset 1 as they represent mostly fishes in the intersex stage [25]. In dataset 2, the stages covering early differentiation, before gametogenesis, (D0 to D30) were chosen as the most relevant in the context studied [29]. Since sexual dimorphic gene expression patterns have been shown to appear from 35 dpf in rainbow trout [34,35], the earliest impact of EE2 on rainbow trout differentiation processes has to be considered.

GO Terms and biological pathways helps interpreting gene expression data in terms of the affected biological processes, pathways and phenotypic changes and on the mode of action and toxicity of a compound [36] including exposure to EDCs [6,37,38]. However especially in fish, namely here the rainbow trout, ORA approaches lacks of resources, genome annotations being scarce and/or heterogeneous. We thus chose to work by gene homology with *Danio rerio*, the only fish for which accurate pathways maps were available on Kegg [39]. A unified annotation of the platforms were made by retrieving *Danio rerio* ensembl gene IDs (ENSDARG) [24]. Considering that many genes and cellular functions are well conserved across taxa [40], the results here can be extrapolated as the general mode of action of EE2 on the juvenile male fish testis transcriptome. In Table 2, 12 pathways and 23 GO Terms appeared as significantly enriched in our results, 3 being significant after the Benjamini correction, shown to be too conservative for the EASE score in previous studies [19,22–24].

### 4.2. Pathways and ontologies associated with EE2 exposure in juvenile fish

We have previously shown that homology with *Danio rerio* retrieved several pathways associated with testis-ova development in juvenile rainbow trout exposed to low doses of EE2 [24]. The meta-analysis also identified numerous pathways as significantly enriched with differentially expressed genes. The advantage of the meta-analysis that it looks at DEGs resulting from EE2 exposure in juvenile fish in several conditions (*i*.*e*. several low and high EE2 doses; short and long exposure time), thus enhancing their relevancy as a fingerprint of EE2 exposure.

The general pattern of pathways and GO Terms obtained with the meta-analysis was similar to that obtained in our previous study [24], with the Cell Cycle and DNA replication being the most significantly enriched pathways, and several others related to Genetic information processing and Metabolism (Nucleotide excision repair, Mismatch repair, RNA degradation, Pyrimidine metabolism, Valine, leucine and isoleucine degradation). This was probably due to the high number of genes common to both analyses. This confirms the pathways proposed by Moggs [36] and Fertuck et al. [41] for E2 and EE2 in their study based on the rodent uterotrophic assay. Two new interesting pathways involved in the Endocrine system, namely the “progesterone-mediated oocyte maturation” and “PPAR signaling” pathways, emerged from this analysis. To our knowledge, this is the first time they have been reported after exposure to EE2 in juvenile fish. Half of the DEGs enriched in the “progesterone-oocyte maturation” pathway are common to the “Cell cycle” and correspond to genes expressed in the germline and involved in cell division (several members of the cyclin family: *ccna1*, *ccnb1*, *ccnb2*, *cdk2; mad2l1*, *anacp5*, *cdc16*) [42]. *ccna1* is a key gene involved in the control of meiosis [42]. Several kinases shared with the oocyte meiosis pathways are also represented (*rps6ka1*, *mos*). They are implicated in the phosphorylation of different substrates in the mitogen-activated protein kinase (MAPK) signaling pathway involved in cell growth and differentiation [43]. Other ubiquitous genes coding for chaperones (*hsp90Ab1*, *hsp90a*.*2*.) or G proteins (*gnai21*, *gnai3*) are represented. They are involved in numerous cellular processes. In view of these results, it is difficult to assess whether the overrepresentation of this pathway is specific to the progesterone-mediated response or is due to the differential expression of ubiquitous genes implicated in cellular processes, and especially cell division. However, during normal male testis development, meiosis occurred later than in females. These early meiotic signatures in males exposed to EE2 might therefore originate from pre-vitellogenic oocyte development. It will be then interesting to study this pathway in greater detail with reference to rainbow trout physiology, once further information becomes available.

The overrepresentation of the peroxisome proliferator-activated receptor (PPAR) signaling pathway is very interesting in the studied context. Peroxisome proliferator-activated receptors (PPARs) are ligand-inducible transcription factors belonging to the nuclear hormone (steroid/thyroid/retinoid) receptor (NHR) superfamily [44]. PPARs regulate gene expression by binding with RXR (Retinoid X Receptor) as a heterodimeric partner to specific DNA sequence elements termed PPREs (Peroxisome Proliferator Response Elements). Target genes are mostly implicated in lipid transport and metabolism [45] and, in recent years, PPARs have emerged as critical regulators of lipid homeostasis in mammals [46]. Moreover, PPARs could play a major role in testicular somatic cells [45]. To date, three PPAR subtypes have been described in mammals and other species: PPARα, PPARβ and PPARγ [47]. They display different tissue distribution and function [46]. In *Xenopus laevis*, oocytes express high levels of PPARβ and smaller amounts of PPARα [44]. Endogenous ligands are mostly fatty acids and their derivatives, but a wide range of various natural and synthetic ligands of PPARs have been described [46]. Several estrogenic compounds (17b-estradiol, NP, genistein) have been shown to interact with PPARs [47–50]. PPARs are also upregulated in testis of rainbow trout treated with T or 11-KT during spermatogenesis [45]. Moreover, both laboratory and field studies have shown that a range of environmental pollutants (including PCBs, alkylphenols and estrogens) provoke peroxisome proliferation in different fish or bivalve mollusc species via the activation of PPARs [51]. Interestingly, the PPAR/RXR heterodimer-responsive element has been characterized in the promoter regions of the zebrafish and *Fundulus heteroclitus cyp19a1* genes [49,52]. This indicates that ligands of PPAR may affect the regulation of aromatase genes and therefore have an impact on the reproductive and developmental processes regulated by E2. It has been shown that several PPAR ligands have an inhibitory effect on aromatase expression in normal human breast adipose tissue [53] and ovarian granulosa cancer cells [54]. All these results argue in favor of a possible alteration of the PPAR signaling pathway by ethynylestradiol, as is suggested by our meta-analysis. Further studies will be necessary to determine whether the alteration observed is directly mediated by EE2, and related to a disruption in lipid metabolism regulation and homeostasis, or is involved in early sex differentiation.

Overall, the GO term enrichment was highly correlated with the pathway analysis, with a majority of terms related to genes involved in cell division. Terms related to sexual reproduction (with an enrichment of genes related to male gamete maturation and spermatogenesis) were more represented and appeared in the top list in terms of significance. The term “Oxidoreductase activity”, which covers genes coding for key enzymes involved in steroidogenesis (*cyp17a1*, *cyp19a1*, *hsd11b2*), was absent in the pathway analysis.

### 4.3. Relevant genes potentially implicated in the early male-to-female transdifferentiation process

The final step of our analysis retrieved 20 statistically and physiologically relevant genes potentially implicated in the male-to-female transdifferentiation process in trout. Indeed, those genes are expressed shortly after EE2 treatment, at early developmental stages (7–16 and 30 days after the treatment, with the fish being at 55 dpf on day 0), and in intersex fish following chronic exposure to low doses of EE2 (namely 0.01 and 0.1 μg/L). Among those genes, ten (*kidins220*, *tekt1*, *gsta*, *hsd17b6*, *aox1*, *lamb1*, *cyp17a1*, *tbx1*, *cyp11b* and *lim2*) were found to be also differentially expressed during normal female development compared to control males, at early developmental stages (D0 to D30 in dataset 2), with a very similar pattern (Fig 5A–5J). Most of these genes were underexpressed, except *gsta* and *hsd17b6*. Only the expression patterns of *kidins220* and *tekt1* were non-similar, as they were overexpressed in dataset 1 and underexpressed in dataset 2. The other ten genes displayed expression patterns specific to the EE2 treatment (*vtg*, *rsph1-3-9*, *pltp*, *foxf1*, *αtub84d*, *p4hb*, *csrp2*, *arlip6*) (Fig 5K–5T). They all showed overexpression compared to control males (except *p4hb* and *csrp2*), with a peak at D30 in dataset 2. Female expression was similar to that of control males (except for p4hb). This indicates that alterations in the expression of genes naturally involved in the early female differentiation process may be involved in the early EE2-induced feminization.

#### 4.3.1. Genes involved in natural early female differentiation

Three of these genes encode key steroidogenic enzymes involved in 11-oxygenated androgen (including 11-KT) biosynthesis in the testis [55]. The 17-α-hydroxylase/17,20 lyase (encoded by *cyp17a1*) converts progesterone into 17α-hydroxyprogesterone and androstenedione through its hydroxylase and lyase activity, respectively. 17β-hydroxylase (encoded by *hsd17b*) then converts androstenedione into testosterone, which in turn is transformed into 11β-hydroxytestosterone, a precursor of 11-KT, by 11β-hydroxylase (encoded by *cyp11b*) [56]. 11-KT is a key androgen involved in testicular differentiation and spermatogenesis in most fishes [57]. In rainbow trout, *cyp11b* and *cyp17a1* are marker genes of early testicular development, and their expression is upregulated in males gonads with a clear sex dimorphism starting at 35 dpf for *cyp11b*, and later (70 dpf) for *cyp17a1* [29,34]. This overexpression in the testis continues throughout male gametogenesis [58,59]. Conversely, *hsd17b* shows marked overexpression during the early oogenesis period (from d60 to d110) and has been shown to be overexpressed during oocyte development [29,57,58]. The alteration of steroidogenesis following exposure to exogenous hormonal treatment or environmental contaminants has been extensively reported in several fish species [8,38,60–63]. In our meta-analysis, *cyp17a* and *cyp11b* were found to be underexpressed under the treatment. Both *in vivo* and *in vitro* studies have shown rapid, strong underexpression of *cyp11b* and *cyp17a* mRNA following E2 or EE2 treatment during and after sex differentiation [8,60,61,63,64], which supports a direct effect of E2 on the testis. This leads to a decrease in circulating androgen levels, as has been measured in several experiments. *Cyp17a1* and *hsd17b* are underexpressed following a short period of exposure to E2 and EE2 in male fathead minnows, whereas their expression is overexpressed in females [60]. Inversely, *hsd17b* was overexpressed in our results, in keeping with its natural overexpression observed in females (Fig 5H). Several types of 17βHSD enzymes exist and their relative roles have not been well defined [65]. Considering that testosterone is the precursor of E2, this enzyme is also implicated in regulating estrogen availability. It is then unclear why this enzyme is overexpressed. However, decreased levels of 11KT were recorded in exposed fish from dataset 1 (article 1). Altogether, the presence of these three genes among our candidates confirms their important role as key regulators of the feminization process, probably through a decrease in androgen levels. More importantly, feminization seems to rely essentially, though not exclusively, on the quick and strong decrease in *cyp11b* expression, whose expression profiles and significance are consistent in all the contrasts considered in our meta-analysis (Fig 3). Moreover, *cyp11b* has already been identified as a key actor triggering the male-to-female transdifferentiation process [8,64].

Another early testicular marker of testis development (*tbx1*) was found among the relevant genes, and was strongly underexpressed in both analyses (dose-dependently in dataset 1 –Fig 4A; and with a peak at D30 in dataset 2- Fig 5D), which is consistent with previous studies showing strong downregulation of tbx1 by EE2 [66]. *tbx1* is a member of the T-box transcription factor gene family involved in embryogenesis and organogenesis, whose expression is restricted to the somatic cells in the testis and appears to be sexually dimorphic very early in differentiating gonads (from 35 dpf)[66,67]. *tbx1* is also expressed in the adult testis, and its expression decreases sharply throughout the reproductive cycle [42,68]. Our results support its role in the control and regulation of testicular differentiation, and suggest the importance of its downregulation during the feminization process.

*lamb1* and *tekt1* encode proteins belonging to the laminin and tektin protein families which are structural macromolecules found in the basal lamina and cytoskeleton, respectively. The basement membrane related gene (*lama5*) has been reported to be involved in gonadal development of both sexes in rat and fish [29]. Genes encoding tektins are predominantly expressed in the testis within the germline and during late spermatogenesis (stage IIIb and V) and are involved in microtubule cytoskeleton organization, ciliary or flagellar mobility [42]. In mice, this gene seems to play a role in spermatogenesis and *tekt1* mRNA are measured in spermatocytes and spermatids within seminiferous tubules [69]. Several other genes sharing similar expression profiles with normal females, have never, to our knowledge, been reported as being directly involved in sex differentiation or feminization. *Aox1* belongs to a highly conserved family of proteins with oxidoreductase activity. The physiological functions and substrates of AOXs are still unknown, although they are recognized as major drug metabolizing enzymes [70]. The expression profiles of the *Lim2* gene are of particular interest, as it was strongly underexpressed in both datasets, with increased repression between D7 and D111. It was reported in the enriched “Organogenesis” GO Term in our previous study [24]. However, this gene encodes the intrinsic Lens fiber membrane protein, with unknown function. *Kidins220* (220 kDa kinase D-interacting substrate) is a substrate of protein kinase D (PKD). This gene encode for a membrane protein mostly expressed in brain and neuroendocrine cells, with no detectable mRNA levels in the rat testis [71]. Finally, glutathione S-transferase A (encoded by *gsta*) belongs to a family of isoenzymes recognized as classical markers of toxicity, due to their well described role in the detoxification of endogenous and exogenous substances [72]. Numerous studies have reported its overexpression (mostly in the liver) following exposure to xenobiotics [73,74]. Further investigation will be necessary to determine why all of these gene expressions are altered during natural early female development, why they respond to EE2 treatment, and their potential role in early sex differentiation.

#### 4.3.2. Genes specific to EE2 exposure

Among those genes, the well-known biomarker of estrogen exposure VTG was selected, which confirms the validity of our analysis to retrieve important genes responding to EE2 treatment. The gene *αtub84d* encoding a cytoskeleton component was found to be overexpressed during spermatogenesis [42] and the gene product of *ARL6IP1* was reported to be involved in protein transport, membrane trafficking or cell signaling during hematopoietic maturation *in vitro* [75,76] and in neural crest development in zebrafish embryos *in vivo* [77]. Its *in vivo* roles during embryonic development are still largely unknown. Three genes encoding members of the radial spoke head protein family (*rsph 1*, *rsph 2* and *rsph 9)* were selected in our meta-analysis. They were found to be upregulated by the EE2 treatment in both datasets, with a peak of expression between D16 and D91 in dataset 2. These proteins are involved in various functions such as flagella motility, sensory functions and development. *rsph3* has been reported among genes preferentially expressed in meiotic and post-meiotic germ cells in the testis of rainbow trout and were upregulated following T supplementation *in vivo* [78]. *p4hb* displays a peculiar pattern of expression, with clear underexpression between D7 and D30 closely related to the female curve. However, numerous studies reported its overexpression in the liver of several fish species, including rainbow trout, following estrogen treatment [79–81], and even proposed it as an effective biomarker of xenoestrogen exposure. A specific profile of expression related to early development in the testis has thus been identified here and must be confirmed. *pltp* encodes for a protein involved in lipid transport in the PPAR signaling pathway. Considering the possible impact of the alteration of this pathway on expression levels of aromatase, the key enzyme that triggers the female developmental processes (see pathway discussion section above), the implication of this pathway in sex (trans)differentiation would constitute a very interesting topic for further study. In view of these results, these gene products do not seem to be directly involved in differentiation processes, or are involved to a still unknown extent. Their expression profiles might be further investigated as biomarkers of estrogen exposure during early differentiation in rainbow trout.

Two other genes in this top list, namely *foxf1* and *csrp2*, are transcription factors whose altered expression can lead to numerous changes in downstream gene transcript expression. *foxf1* belongs to the family of forkhead transcription factors, known as key players in development and metabolism (*foxl2* is a important gene triggering female differentiation and development in fish) [82]. *foxf1* expression during early development in rainbow trout is located mainly in the digestive tract and gills, and is associated with the development of gill filaments and lamellae [83]. The presence of ERE sequences in the promoter region of several fox genes (including *foxf1*) suggests that their transcriptional regulation is under ER control [84]. This is supported, at least for *foxf1*, by our results showing overexpression of this gene in both datasets. Cysteine and glycine-rich protein 2 (*csrp2*) is involved in cell differentiation. Its expression is naturally dimorphic during early differentiation, and it is underexpressed in females. Following EE2 treatment, it appears as strongly repressed in dataset 1 and to a lesser extent in dataset 2. This repression of *csrp2* expression was also reported after exposure to sewage in Chinook salmon (*Oncorhynchus tshawytscha*) [85].

## Conclusions and Perspectives

In conclusion, our bioinformatics workflow meta-analysis successfully retrieved pathways related to estrogen exposure in fish and suggested novel ones. This led to the formulation of new hypotheses to be tested in order to elucidate the mechanisms underlying the modes of actions of estrogen, particularly in juvenile fish. Comparative homology analysis with *Danio rerio* thus appeared to be a relevant and powerful approach and suggests that its use could be extended to other fish species for environmental risk assessment purposes. Moreover, our approach identified several genes potentially implicated in the early trans-differentiation process. The expression of half of these genes was closely related to genes differentially expressed during natural female differentiation, including several markers repressed during testis development (*cyp11b*, *cyp17a1*, *tbx1*). Of particular interest is the quick and strong underexpression of *cyp11b*, which encodes the enzyme responsible for androgen production, which appears to be a key step in the feminization process. Other genes (including VTG) were found to respond specifically to the estrogen treatment. This underscores that EE2 acts by different mechanisms to trigger feminization induction in males, some closely related to normal mechanisms in developing females, other triggering the expression of genes not usually expressed in a natural feminization process. Although the expression of several of these genes has been verified on *in vitro* models (*i*.*e*. *cyp11b* in rainbow trout [63], *cyp17a1* in zebrafish [86], *VTG* in numerous species, including rainbow trout [87], suggesting a direct response to EE2), these results must still be validated to assess whether the altered expression of all of these genes is the result of a direct steroid action of the estrogen on the testis, or reflects an indirect retro control on the hypothalamus–pituitary axis, or a conjunction of both.

## Supporting Information

S1 Dataset Pre-processed data of dataset 2. (XLSX)

S1 Results DAVID software output from Intersection I DEG group analysis. Exhaustive list of pathways obtained with Intersection I DEG group from the meta-analysis between dataset 1 and dataset 2.
(DOCX)

S2 Results EASE software output from Intersection II DEG group analysis. Exhaustive (p<0.01) list of ontologies obtained with Intersection II DEG group from the meta-analysis between dataset 1 and dataset 2. Genes belonging to each GO term are represented.
(XLSX)
