# Supplementary material for: Meta-Analysis of Microarray Data of Rainbow Trout Fry Gonad Differentiation Modulated by Ethynylestradiol
Source: PLoS One. 2015 Sep 17;10(9):e0135799. doi: 10.1371/journal.pone.0135799 (PMC4574709; doi:10.1371/journal.pone.0135799)
Supplement: Supplementary file 1 [file pone.0135799.s002.docx]

DAVID Bioinformatics Resources 6.7
National Institute of Allergy and Infectious Diseases (NIAID), NIH

Functional Annotation Chart

Help and Manual

Current Gene List: List_1
Current Background: Background_2
1171 DAVID IDs

Options

Rerun Using Options | Create Sublist

79 chart records

Download File

Sublist	Category	Term	RT	Genes	Count	%	P-Value	Benjamini
	KEGG_PATHWAY	Cell cycle	RT		34	2,9	1,1E-6	1,3E-4
	KEGG_PATHWAY	DNA replication	RT		15	1,3	2,9E-5	1,8E-3
	KEGG_PATHWAY	Oxidative phosphorylation	RT		29	2,5	6,0E-5	2,5E-3
	KEGG_PATHWAY	Pyrimidine metabolism	RT		20	1,7	3,7E-3	1,1E-1
	KEGG_PATHWAY	Nucleotide excision repair	RT		12	1,0	8,2E-3	1,8E-1
	KEGG_PATHWAY	Mismatch repair	RT		8	0,7	1,1E-2	2,0E-1
	KEGG_PATHWAY	Fatty acid elongation in mitochondria	RT		5	0,4	1,5E-2	2,4E-1
	KEGG_PATHWAY	Ribosome	RT		18	1,5	1,6E-2	2,2E-1
	KEGG_PATHWAY	PPAR signaling pathway	RT		12	1,0	3,4E-2	3,8E-1
	KEGG_PATHWAY	RNA degradation	RT		12	1,0	4,4E-2	4,2E-1
	KEGG_PATHWAY	Progesterone-mediated oocyte maturation	RT		16	1,4	4,7E-2	4,2E-1
	KEGG_PATHWAY	Valine, leucine and isoleucine degradation	RT		10	0,9	4,8E-2	3,9E-1
	KEGG_PATHWAY	p53 signaling pathway	RT		12	1,0	8,5E-2	5,6E-1
	KEGG_PATHWAY	Cardiac muscle contraction	RT		13	1,1	8,6E-2	5,4E-1
	KEGG_PATHWAY	Oocyte meiosis	RT		17	1,5	1,1E-1	6,0E-1
	KEGG_PATHWAY	Purine metabolism	RT		22	1,9	1,1E-1	5,9E-1
	KEGG_PATHWAY	Citrate cycle (TCA cycle)	RT		7	0,6	1,6E-1	7,0E-1
	KEGG_PATHWAY	Glutathione metabolism	RT		7	0,6	1,9E-1	7,6E-1
	KEGG_PATHWAY	Protein export	RT		3	0,3	2,0E-1	7,6E-1
	KEGG_PATHWAY	Galactose metabolism	RT		5	0,4	2,1E-1	7,6E-1
	KEGG_PATHWAY	Arachidonic acid metabolism	RT		6	0,5	2,3E-1	7,9E-1
	KEGG_PATHWAY	Glycosphingolipid biosynthesis	RT		3	0,3	2,5E-1	7,9E-1
	KEGG_PATHWAY	Propanoate metabolism	RT		6	0,5	2,8E-1	8,3E-1
	KEGG_PATHWAY	Homologous recombination	RT		5	0,4	2,9E-1	8,2E-1
	KEGG_PATHWAY	Selenoamino acid metabolism	RT		5	0,4	2,9E-1	8,2E-1
	KEGG_PATHWAY	Base excision repair	RT		6	0,5	3,1E-1	8,3E-1
	KEGG_PATHWAY	Fructose and mannose metabolism	RT		7	0,6	3,2E-1	8,3E-1
	KEGG_PATHWAY	Renin-angiotensin system	RT		3	0,3	3,4E-1	8,5E-1
	KEGG_PATHWAY	Biosynthesis of unsaturated fatty acids	RT		4	0,3	3,6E-1	8,6E-1
	KEGG_PATHWAY	Nitrogen metabolism	RT		4	0,3	3,6E-1	8,6E-1
	KEGG_PATHWAY	ECM-receptor interaction	RT		9	0,8	3,7E-1	8,5E-1
	KEGG_PATHWAY	Arginine and proline metabolism	RT		8	0,7	3,9E-1	8,6E-1
	KEGG_PATHWAY	Glycolysis / Gluconeogenesis	RT		9	0,8	3,9E-1	8,5E-1
	KEGG_PATHWAY	Drug metabolism	RT		5	0,4	4,0E-1	8,6E-1
	KEGG_PATHWAY	Cysteine and methionine metabolism	RT		6	0,5	4,1E-1	8,5E-1
	KEGG_PATHWAY	Pyruvate metabolism	RT		6	0,5	4,3E-1	8,7E-1
	KEGG_PATHWAY	Proteasome	RT		7	0,6	4,5E-1	8,8E-1
	KEGG_PATHWAY	Taurine and hypotaurine metabolism	RT		2	0,2	4,7E-1	8,8E-1
	KEGG_PATHWAY	One carbon pool by folate	RT		3	0,3	4,7E-1	8,8E-1
	KEGG_PATHWAY	Limonene and pinene degradation	RT		3	0,3	4,7E-1	8,8E-1
	KEGG_PATHWAY	Glycerolipid metabolism	RT		5	0,4	5,1E-1	9,0E-1
	KEGG_PATHWAY	Fatty acid metabolism	RT		5	0,4	5,1E-1	9,0E-1
	KEGG_PATHWAY	Cell adhesion molecules (CAMs)	RT		10	0,9	5,3E-1	9,0E-1
	KEGG_PATHWAY	Steroid hormone biosynthesis	RT		4	0,3	5,6E-1	9,2E-1

Sublist	Category	Term	RT	Genes	Count	%	P-Value	Benjamini
☐	KEGG_PATHWAY	Glycine, serine and threonine metabolism	RT		4	0,3	5,6E-1	9,2E-1
☐	KEGG_PATHWAY	RNA polymerase	RT		4	0,3	5,6E-1	9,2E-1
☐	KEGG_PATHWAY	VEGF signaling pathway	RT		8	0,7	5,7E-1	9,2E-1
☐	KEGG_PATHWAY	Amino sugar and nucleotide sugar metabolism	RT		6	0,5	5,9E-1	9,3E-1
☐	KEGG_PATHWAY	NOD-like receptor signaling pathway	RT		6	0,5	6,2E-1	9,3E-1
☐	KEGG_PATHWAY	mTOR signaling pathway	RT		6	0,5	6,4E-1	9,4E-1
☐	KEGG_PATHWAY	Natural killer cell mediated cytotoxicity	RT		8	0,7	6,4E-1	9,4E-1
☐	KEGG_PATHWAY	Cytosolic DNA-sensing pathway	RT		4	0,3	6,4E-1	9,3E-1
☐	KEGG_PATHWAY	Basal transcription factors	RT		4	0,3	6,4E-1	9,3E-1
☐	KEGG_PATHWAY	Lysosome	RT		12	1,0	6,6E-1	9,4E-1
☐	KEGG_PATHWAY	Butanoate metabolism	RT		4	0,3	6,7E-1	9,4E-1
☐	KEGG_PATHWAY	N-Glycan biosynthesis	RT		5	0,4	6,8E-1	9,4E-1
☐	KEGG_PATHWAY	Adipocytokine signaling pathway	RT		7	0,6	7,1E-1	9,5E-1
☐	KEGG_PATHWAY	Androgen and estrogen metabolism	RT		3	0,3	7,1E-1	9,5E-1
☐	KEGG_PATHWAY	Spliceosome	RT		13	1,1	7,1E-1	9,5E-1
☐	KEGG_PATHWAY	Pentose and glucuronate interconversions	RT		2	0,2	7,2E-1	9,5E-1
☐	KEGG_PATHWAY	Toll-like receptor signaling pathway	RT		8	0,7	7,3E-1	9,5E-1
☐	KEGG_PATHWAY	Alanine, aspartate and glutamate metabolism	RT		4	0,3	7,3E-1	9,5E-1
☐	KEGG_PATHWAY	Apoptosis	RT		8	0,7	7,6E-1	9,5E-1
☐	KEGG_PATHWAY	Glycosphingolipid biosynthesis	RT		2	0,2	7,8E-1	9,6E-1
☐	KEGG_PATHWAY	Glyoxylate and dicarboxylate metabolism	RT		2	0,2	7,8E-1	9,6E-1
☐	KEGG_PATHWAY	Tryptophan metabolism	RT		4	0,3	7,9E-1	9,6E-1
☐	KEGG_PATHWAY	RIG-I-like receptor signaling pathway	RT		5	0,4	8,2E-1	9,7E-1
☐	KEGG_PATHWAY	TGF-beta signaling pathway	RT		8	0,7	8,3E-1	9,7E-1
☐	KEGG_PATHWAY	Notch signaling pathway	RT		5	0,4	8,3E-1	9,7E-1
☐	KEGG_PATHWAY	Riboflavin metabolism	RT		2	0,2	8,3E-1	9,7E-1
☐	KEGG_PATHWAY	Starch and sucrose metabolism	RT		3	0,3	8,3E-1	9,7E-1
☐	KEGG_PATHWAY	Steroid biosynthesis	RT		2	0,2	8,5E-1	9,7E-1
☐	KEGG_PATHWAY	Inositol phosphate metabolism	RT		5	0,4	8,6E-1	9,8E-1
☐	KEGG_PATHWAY	Regulation of autophagy	RT		2	0,2	8,7E-1	9,8E-1
☐	KEGG_PATHWAY	Pentose phosphate pathway	RT		3	0,3	8,9E-1	9,8E-1
☐	KEGG_PATHWAY	ErbB signaling pathway	RT		7	0,6	8,9E-1	9,8E-1
☐	KEGG_PATHWAY	Heparan sulfate biosynthesis	RT		2	0,2	9,0E-1	9,8E-1
☐	KEGG_PATHWAY	Glycosylphosphatidylinositol(GPI)-anchor biosynthesis	RT		2	0,2	9,0E-1	9,8E-1
☐	KEGG_PATHWAY	Phenylalanine metabolism	RT		2	0,2	9,0E-1	9,8E-1

843 gene(s) from your list are not in the output.

Please cite *Nature Protocols 2009; 4(1):44 & Genome Biology 2003; 4(5):P3* within any publication that makes use of any methods inspired by **DAVID**.

Term of Service | Contact Us | Site Map
